# Profiles of disability among youths in Singapore and their link to psychological distress and health care utilization

**DOI:** 10.3389/fpsyt.2026.1821187

**Published:** 2026-06-01

**Authors:** Edimansyah Abdin, Bernard Tan, Sherilyn Chang, Ellaisha Samari, Brian Tan, Charmaine Tang, Janhavi Vaingankar, Swapna Verma, Mythily Subramaniam

**Affiliations:** 1 Research Division, Institute of Mental Health, Singapore, Singapore; 2 Department of Psychosis, Institute of Mental Health, Singapore, Singapore; 3 Medical Board, Institute of Mental Health, Singapore, Singapore; 4 Saw Swee Hock School of Public Health, National University of Singapore, Singapore, Singapore

**Keywords:** DASS-21, disability, distress, healthcare utilisation, latent class analyses, Singapore, WHODAS 2.0, youth

## Abstract

**Background/objectives:**

Disability in youth is an increasing global public health concern, with growing evidence linking disability to mental disorders and elevated healthcare needs. This study aimed to identify disability subtypes among Singaporean youth, examine sociodemographic factors, and assess their relationships with psychological distress and healthcare utilization.

**Methods:**

Data were drawn from the National Mental Health Youth Study (n = 2,600), a cross sectional epidemiological survey of Singapore residents aged 15-35 years. Latent class analysis was conducted using 12 indicator variables from the World Health Organization Disability Assessment Schedule 2.0. Multinomial and multivariable logistic regression models examined sociodemographic correlates and associations with psychological distress and healthcare utilization.

**Results:**

A four−class solution best represented disability patterns: high difficulty (7.8%), moderate social and functional difficulty (13.8%), high physical and cognitive difficulty (6.7%), and no/low difficulty (71.7%). Youth in the high difficulty class were more likely to be of Malay and Indian ethnicity and have lower educational attainment. Compared with the no/low difficulty class, the high difficulty class had higher odds of moderate (OR=3.2) and severe/very severe depression (OR=4.1), as well as moderate (OR=2.2) and severe/very severe anxiety (OR=3.6). Youth in the high difficulty and moderate social and functional classes also had higher odds of hospital admissions, A&E visits, and contact with polyclinic and restructured hospital doctors.

**Conclusion:**

Distinct disability profiles among Singaporean youth are strongly linked to psychological distress and higher healthcare use, highlighting the need for earlier identification and targeted interventions to better support youths experiencing functional difficulties and psychological distress.

## Introduction

Disability in youth is a growing public health concern worldwide. Previous studies have shown a substantial rise in the numbers of children and adolescents living with disability, with conditions such as migraine, asthma, hearing impairment or injuries now affecting over 100 million individuals among those aged 20 years and below (1). A systematic analysis of the Global Burden of Disease (GBD) study has also shown that more than 15% of disability-adjusted life years (DALYs) were due to adolescents and young adults (2). The World Health Organization (WHO) (3) has reported that it is important to move beyond a simple medical diagnosis when addressing disability by adopting a broader understanding that includes difficulties in functioning and participation in daily life. The WHO’s International Classification of Functioning, Disability and Health (ICF) provides a biopsychosocial framework for this purpose. It defines disability as a dynamic interaction between health conditions and environmental and personal factors (4). Based on this framework, people can be classified through a detailed description of their functioning across key domains such as impairments of body structure, limitations in activities, and restrictions in participation (4).

The World Health Organization Disability Assessment Schedule (WHODAS 2.0) has been developed to operationalize this framework into an assessment tool that can measure six key domains of disability including mobility, self-care, getting along, life activities, participation, and cognition (5). This perspective is crucial for young people, as functional limitations during this crucial period can influence educational attainment, social integration, and the transition to adulthood, with long-term consequences for their well-being and economic productivity (6).

A person-centred approach, such as Latent Class Analysis (LCA) (7), is essential for uncovering distinct subgroups of individuals with similar patterns of functional difficulties, providing a more precise characterization of disability than a simple severity score. This method is empirically able to detect unobserved (latent) subgroups of individuals, where each subgroup is characterized by a shared probabilistic responses pattern across multiple functional domains (8, 9). The usefulness of this statistical approach has been reported in previous studies among youth, including studies on youth’s daily coping strategies (10), physical and sexual abuse (11), and family characteristics linked to youth offending outcomes (12). Furthermore, a significant association has been reported between mental disorders and disability. Mental disorders were found to be strongly associated with severe disability (13). However, previous studies have primarily used variable-centred approaches to analyse disability, with relatively few examining this relationship using person-centred disability profiles through latent class analysis.

Singapore is a city-state in Southeast Asia with a diverse, multiethnic population, predominantly Chinese (74%), followed by Malays (13.5%), Indians (9%), and other ethnic groups (3.4%). In Singapore, the majority (35.7%) of registered persons with disabilities are reported to be aged between 19 and 34 years old, which is the highest proportion among all age groups (14). Although youth represent the largest proportion of registered persons with disabilities, there is limited understanding of specific disability profiles within this population based on the ICF framework, as well as their relationship to psychological distress and healthcare utilization. Hence, the purpose of this study is to identify disability subtypes among Singaporean youth, determine the associated sociodemographic factors, and examine their links to psychological distress and healthcare utilization.

## Methods

### Study design and participants

This study used data from a cross-sectional epidemiological survey of Singapore residents aged 15–35 years. The study methodology was reported in detail previously (15). Participants were literate in English, Mandarin, Malay, or Tamil and provided written informed consent. For participants below 21 years of age, consent was also obtained from a legally acceptable representative. The study was conducted in accordance with the principles of the Declaration of Helsinki. Ethical approval was obtained from the National Healthcare Group (NHG) Domain Specific Review Board (DSRB).

## Measures

### Disability

The 12-item self-administered version of the WHODAS 2.0 was used to assess 6 domains of disability including cognitive, mobility, self-care, getting along, life activities, and participation. Each item is rated on a 5-point Likert-type scale to reflect the level of difficulty, starting with ‘no difficulty’ and increasing in an ordered fashion from ‘mild,’ ‘moderate,’ ‘severe’ to ‘extreme or cannot do.’ A total score can be calculated by assigning each of the items a score ranging from 0 (mild) −4 (extreme or cannot do) – which are then summed up with total scores ranging from 0 to 48. The reliability and validity of the instrument has been established in Singapore (16). In this study, WHODAS 2.0 items were not treated as a continuous dependent variable, but rather used to identify empirically derived, person-centred disability profiles using LCA. LCA is a well-established analytic approach for uncovering heterogeneous subgroups within a population based on response patterns across multiple indicators (7, 17). When applied to the WHODAS 2.0 items, this method is intended to identify unobserved subgroups characterized by distinct patterns of functional difficulties, rather than to decompose a single severity dimension. This approach has been used in previous studies (8, 9), where disability profiles served as the primary classification variable.

### Psychological distress

The Depression, Anxiety, and Stress Scale (DASS-21) was used to measure symptoms of depression, anxiety, and stress (18). It contains three seven-item subscales assessing symptoms of depression, anxiety, and stress in the past week rated on a 4-point Likert scale (from 0 = ‘did not apply to me’ to 3 = ‘applied to me very much’). The depression, anxiety, and stress subscale scores can be calculated by summing the scores of the items of each subscale and multiplying them by 2 as per the scoring guidelines. Sum scores for each of the subscales range between 0 and 42. Relevant cut-off scores were applied for the depression, anxiety, and stress subscales to indicate normal, mild, moderate, severe and extremely severe levels (18). The reliability and validity of the instrument has been established in Singapore (19). In the current study, psychological distress was treated as an outcome associated with disability class membership, rather than as an independent predictor variable. Respondents reporting symptoms across multiple domains were analysed within each subscale separately, in accordance with established DASS-21 scoring guidelines and its factor structure (18). This approach is theoretically and empirically supported, as depression, anxiety, and stress represent related but distinct dimensions of psychological distress that frequently co-occur and may differentially relate to disability (18–21).

### Healthcare utilization

Healthcare utilization data was obtained using the adapted version of the Client Service Receipt Inventory (CSRI) (22). Respondents were asked whether they had contacted or been admitted to healthcare services in the past three months, using Yes/No response option. In this study, we focussed on five types of healthcare services including public primary care doctors (polyclinic doctor), restructured hospital doctors (a public hospital doctor, which is wholly owned by the government), private hospital/clinic doctors [e.g. general practitioners (GPs)], hospitalization in the government/restructured or private hospitals, and admissions to accident and emergency (A&E) department.

### Sociodemographic variables

Data on age, gender, ethnicity, marital status, education, employment status, and household income was collected using a structured questionnaire.

### Statistical Analysis

Latent Class Analysis (LCA), implemented in Mplus version 8.0, was used to identify empirical profiles of disability based on responses to the 12 items of the WHODAS 2.0. Unlike exploratory factor analysis (EFA), which is a variable-centred technique that identifies latent dimensions underlying inter-item correlations, LCA is a person-centred technique that identifies unobserved subgroups of individuals sharing similar patterns of item endorsement across measured domains, thereby classifying persons rather than items. When applied to the WHODAS 2.0 items, this method was intended to identify unobserved subgroups characterized by distinct patterns of functional difficulties, rather than to decompose a single severity dimension. This approach has been well-established in the literature (8, 9), where disability profiles served as the primary classification variable. For the purpose of LCA, the original items scored on a five-point scale were dichotomized into a binary scoring (0 = no-to-mild difficulty, 1 = moderate-to-extreme difficulty) (8, 9). Models ranging from two to six classes were compared. The optimal number of classes was determined based on a combination of statistical fit indices (Akaike Information Criterion [AIC], Bayesian Information Criterion [BIC], and adjusted BIC), conceptual interpretability, Vuong-Lo-Mendell-Rubin (VLMR) test, and the Lo-Mendell-Rubin adjusted likelihood ratio (LMR) test. Subsequent analyses were employed using multinomial logistic regression to examine sociodemographic factors associated with disability class memberships as well as the associations of disability class membership with psychological distress. Finally, a series of multivariable logistic regression models were performed to assess the association between disability class membership and various healthcare utilization with adjustment for sociodemographic variables.

## Results

A total of 2600 youths completed the survey. The socio-demographic characteristics of the sample is presented in **Table 1**. The sample consisted of 50.2% females and 49.8% males with a mean age of 25.7 years (SD=6.0; range=15-35 years). The majority were Chinese (71.5%), followed by Malays (16.4%), Indians (9.1%), and other (3.1%) ethnicities. Fit statistics for the LCA models ranging from 2 to 6 classes are shown in **Supplementary Table 1**. The LCA identified a 4-class solution as the best representation of disability profiles in the sample. Although the 6-class model yielded the lowest AIC, BIC, and sample size-adjusted BIC values, class enumeration in the LCA requires considering a combination of statistical fit indices, formal likelihood ratio tests, and substantive interpretability. Specifically, the VLMR and LMR likelihood ratio tests, which evaluate whether a k-class model provides significantly better fit than a (k−1)-class model, yielded non-significant results for both the five- and six-class solutions. This indicates neither model offered meaningful statistical improvement over the four-class model. Furthermore, the five- and six-class solutions produced classes that were poorly differentiated and lacked clear conceptual interpretability. The four-class solution was therefore selected as the optimal model.

**Table 1:** Descriptive statistics of the sample.

Variables	N (sample)	Weighted %
Age		
15–19	632	18.8
20–24	672	21.2
25–29	634	25.4
30–35	662	34.6
Gender		
Male	1381	49.8
Female	1219	50.2
Ethnicity		
Chinese	1313	71.5
Malay	658	16.4
Indian	506	9.1
Others	123	3.1
Currently married		
No	2066	76.7
Yes	534	23.3
Employment		
Employed	1461	62.3
Unemployed	166	6.4
*Economically inactive	973	31.3
Monthly Household income		
Below S$5000	974	31.4
S$5000 to S$9,999	859	33.8
S$10,000 to S$19999	550	25
S$20,000 and above	217	9.8
Education		
Primary and below	182	5.1
Secondary	520	16.4
Post-secondary	1255	43.6
University or higher	643	34.8

* Economically Inactive: Homemakers and Students

### Profiles of disability

**Figure 1:**
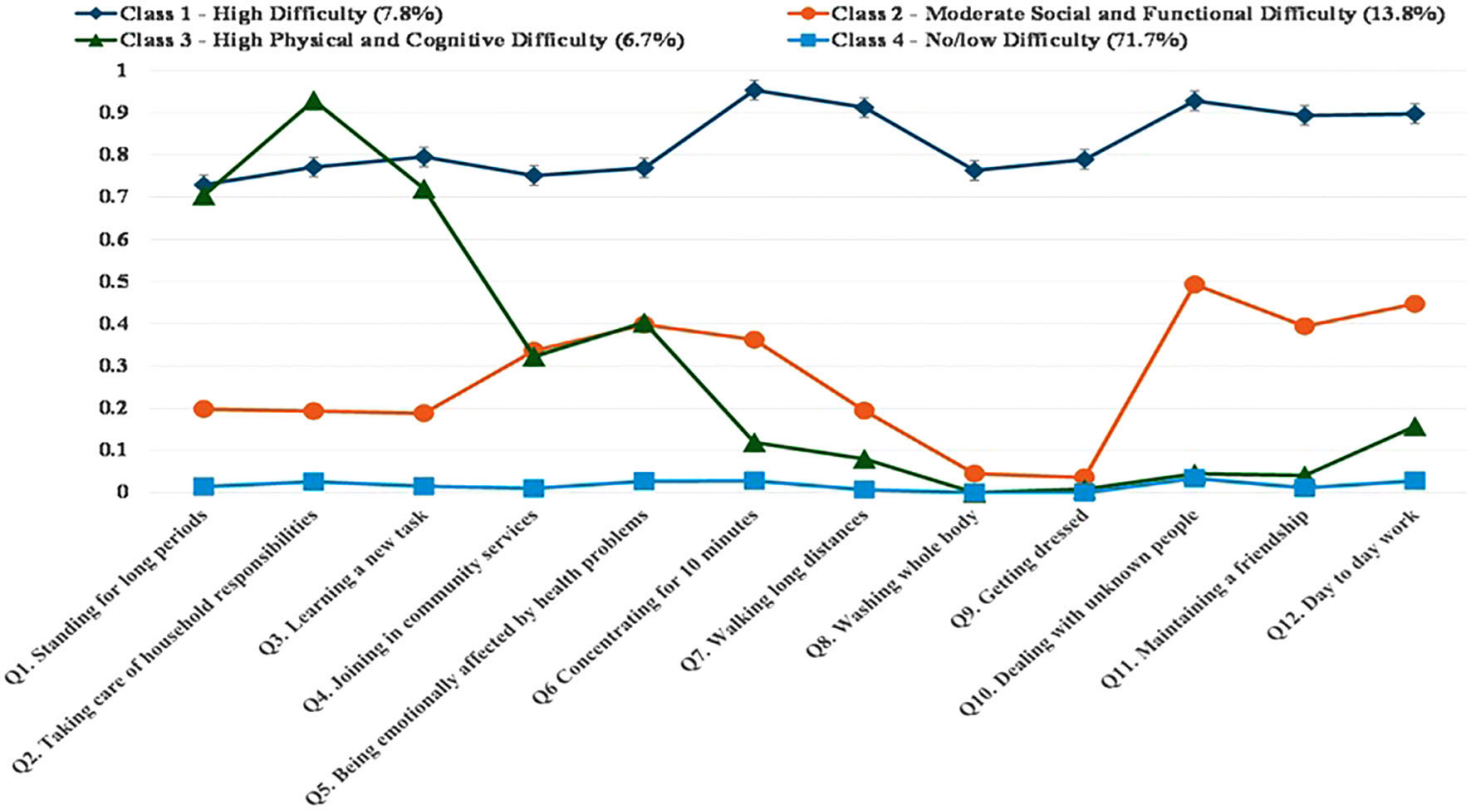
The item response probabilities of disabilities for the four-class solution among youths in Singapore.

Item response probabilities of disabilities for the four-class model are shown in **Supplementary Table 2**. In the 4-class solution (**Figure 1**) , Class 1 (7.8%) exhibited higher probabilities of endorsing moderate-to-extreme difficulty across all items, Class 2 (13.8%) showed moderate probabilities of endorsing moderate-to-extreme difficulty in the items related to dealing with unknown people, maintaining a friendship, and day to day work or school; Class 3 (6.7%) exhibited higher probabilities of endorsing moderate-to-extreme difficulty in items related to problems with household responsibilities, prolonged standing, and task learning. Finally, Class 4 (71.7%), exhibited lower probabilities of endorsing moderate-to-extreme difficulty across all items. To summarize these patterns, Class 1 is termed high difficulty; Class 2, moderate social and functional difficulty; Class 3, high physical and cognitive difficulty; and Class 4, no/low difficulty (**Supplementary Table 2**).

To identify dominant items within each class, we applied a conditional probability threshold of ≥0.30 (i.e., items with a ≥30% probability of moderate-to-extreme difficulty endorsement). Based on this criterion, Class 1 showed high endorsement across all 12 items (all probabilities >0.70); Class 2 showed dominant endorsement for items related to dealing with unknown people (Q10), maintaining a friendship (Q11), and day-to-day work (Q12); Class 3 showed dominant endorsement for prolonged standing (Q1), household responsibilities (Q2), and learning new tasks (Q3); and Class 4 showed uniformly low probabilities (< 0.30) across all items (**Supplementary Table 2**).

### Sociodemographic correlates of disability subtypes

**Figure 2:**
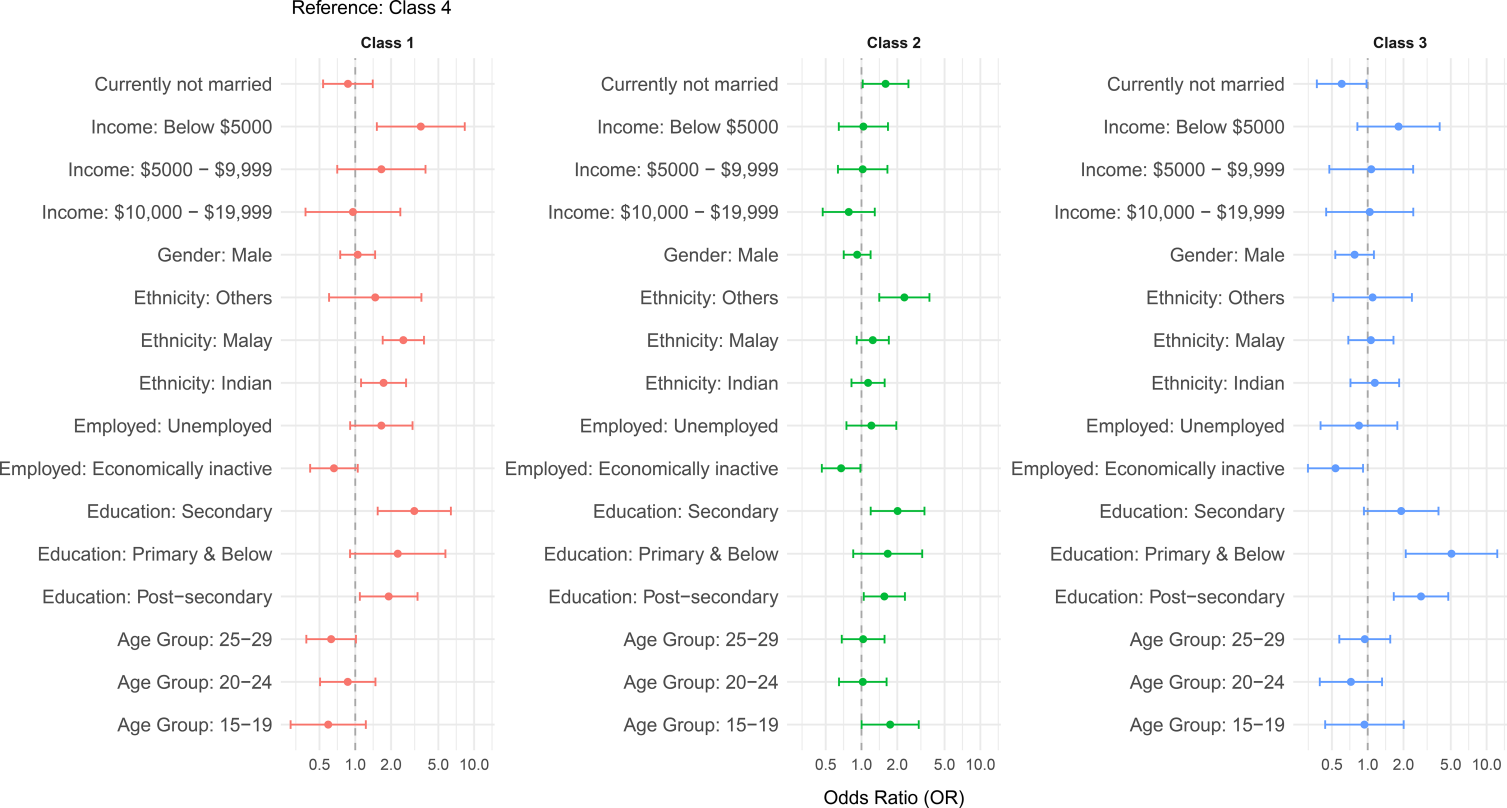
Sociodemographic correlates of disability subtypes among youths in Singapore.

When compared to the no/low difficulty class membership (Class 4), several sociodemographic factors were significantly associated with other class memberships (**Figure 2**). Those of Malay (OR=2.6), and Indian (OR=1.7) ethnicity (vs. Chinese), and those with lower educational attainment (secondary: OR=3.0; post-secondary: OR=1.9) (vs. university or higher) and monthly household income (below $5000: OR=3.6) (vs. $20,000 and above) were more likely to be associated with the high difficulty class membership (Class 1). Those of other (OR=2.4) ethnicity, and those aged 15-19 years (vs. aged 30-35) (OR=1.8), currently not married (OR=1.6) (vs. currently married), and those with lower education (secondary: OR=1.9; post-secondary: OR=1.5) were more likely to be associated with the moderate social and functional difficulty class membership (Class 2). In contrast, those who were economically inactive (OR=0.6) (vs. employed) were less likely to be in the Class 2 membership. We also found that those with lower educational attainment (primary and below: OR=4.6; post-secondary: OR=2.5) were more likely to be associated with the high physical and cognitive difficulty class membership (Class 3), while those who were currently not married (OR=0.6) were less likely to be in the Class 3 membership.

### Association with psychological distress

A strong relationship was observed between disability class membership and psychological distress (**Table 2**). Compared to individuals in the no/low difficulty class membership (Class 4), those with the high difficulty class membership (Class 1) were more likely to be associated with moderate (OR=3.2) and severe/very severe depression (OR=4.1), as well as moderate (OR=2.2) and severe/very severe anxiety (OR=3.6). Those with moderate social and functional difficulty class membership (Class 2) were more likely to be associated with the moderate (OR=2.9) and severe/very severe depression (OR=4.0), mild (OR=2.1), moderate (OR=2.4) and severe/very severe anxiety (OR=2.7), and moderate stress (OR=2.3). Additionally, those with high physical and cognitive difficulty class membership (Class 3) were more likely associated with moderate anxiety (OR=1.7) and severe/very severe stress (OR=2.8).

**Table 2:** Relationship between psychological distress and disability class membership.

Outcome variables	Class 1 – high difficulty	Class 2 – moderate social and functional difficulty	Class 3 – high physical and cognitive difficulty
	OR (95% CI)	OR (95% CI)	OR (95% CI)
DASS-Depression			
Mild	1.4 (0.7,2.9)	0.7(0.2,2.0)	1(0.6,1.9)
Moderate	3.2 (1.9,5.4) *	2.9(1.7,5.2) *	1.2(0.7,2.1)
Severe/Very Severe	4.1 (2.1,8) *	4(2.1,7.6) *	1.9(0.9,3.9)
DASS-Anxiety			
Mild	1.4(0.9,2.2)	2.1(1.2,3.8) *	1.6(0.9,2.4)
Moderate	2.2(1.4,3.5) *	2.4(1.5,3.8) *	1.7(1.04,2.8) *
Severe/Very Severe	3.6(2.2,6.1) *	2.7(1.6,4.8) *	1.6(0.9,2.9)
DASS-Stress			
Mild	0.8 (0.4,1.5)	1.7(0.8,3.6)	0.8(0.4,1.6)
Moderate	1.1 (0.5,2.2)	2.3(1.3,4.1) *	1.6(0.7,3.5)
Severe/Very Severe	2.1 (0.9,4.7)	1.3(0.6,2.9)	2.8(1.3,6.1) *

*p value < 0.01. OR was derived from multiple logistic regression analyses after controlling for sociodemographic variables.

### Association with healthcare utilization

After adjusting for sociodemographic variables, disability class memberships were significantly associated with various forms of healthcare utilization (**Table 3**). Compared to the no/low difficulty class (Class 4), those in Class 1, and 2 were more likely to be admitted in hospital, have A&E visits, and to have contact with the polyclinic doctor, and restructured hospital doctor.

**Table 3:** Relationship between disability class membership and healthcare utilization.

Main predictor variable	Hospitalization	ED	Polyclinic doctor	Restructured hospital doctor	Private doctor
	OR (95% CI)	OR (95% CI)	OR (95% CI)	OR (95% CI)	OR (95% CI)
Disability subtypes					
Class 1 – High Difficulty	3.2 (1.3,7.6) *	2.1 (1.2,3.9) *	1.7 (1.2,2.4) *	1.9 (1.2,3.0) *	1.0 (0.7,1.5)
Class 2 – Moderate Social and Functional Difficulty	3.1 (1.4,7.0) *	3.0 (1.9,4.7) *	1.6 (1.2,2.0) *	2.0 (1.4,2.7) *	1.3 (0.9,1.7)
Class 3 – High Physical and Cognitive Difficulty	0.7 (0.1,3.8)	1.4 (0.6,3.4)	1.1 (0.7,1.6)	1.2 (0.7,2.0)	1.5 (1.0,2.2)
Class 4 – No/Low Difficulty (Reference)					

*p value < 0.01. OR was derived from multiple logistic regression analyses after controlling for sociodemographic variables.

## Discussion

In this study, we used a person-centred approach to identify distinct disability classes among youth in Singapore. Our findings reveal four meaningful subgroups of disability profiles. The majority, comprising 71.7% of the sample, belonged to the ‘no/low difficulty’ class. However, nearly 30% of youth fell into one of three other disability classes, including the ‘high difficulty’ (7.9%), ‘moderate social and functional difficulty’ (14.0%), and ‘high physical and cognitive difficulty’ (6.4%). Our classification of four disability profiles aligns with the previous studies, though the characterization of individual classes may vary slightly. For instance, Macleod et al. (9) identified four subgroups labelled as ‘pervasive disability’, ‘physical disability’, ‘emotional, cognitive, or interpersonal disability’, and ‘no/low disability’. Their ‘pervasive disability’ class is broadly similar to our “high difficulty” class, as both were characterised by having a high likelihood of endorsing all items in the WHODAS 2.0 scale. Similarly, Seet et al. (8) in a recent study among adult patients with schizophrenia, depression, anxiety and diabetes, found five classes of disability, including ‘extensive difficulty’, ‘cognitive and social difficulty’, ‘physical difficulty’, ‘emotional difficulty’, and ‘no difficulty’. Their ‘extensive difficulty’ class was broadly consistent with our “high difficulty” class due to the shared characteristics of high endorsement across all items in the WHODAS 2.0 scale. These consistent findings, identifying a ‘high difficulty’ or ‘pervasive disability’ or ‘extensive difficulty’ subgroup, strongly suggest that certain individuals tend to experience very severe or significant levels of impairment and widespread problems across multiple domains of disability due to their underlying health or mental health conditions. This underscores the critical importance of early identification to facilitate timely and effective treatment, targeted intervention, and rehabilitation strategies among this group (1, 23).

We found that those belonging to the “high difficulty” class were more likely to be of Malay and Indian ethnicity, and had lower education attainment. While our study focuses on youth, these ethnic differences in disability are consistent with previous research that was observed among older adults in Singapore. For instance, Mahesh et al. (24) observed significantly higher disability scores among Indian and Malay participants compared to Chinese participants in a Singaporean elderly population. Furthermore, Ng et al. (25) also found similar ethnic disparities in disability using ADL and IADL measures, suggesting that a higher prevalence of chronic medical illness, could contribute to Malays experiencing higher levels of functional disability compared to the Chinese [24; 25. The disproportionate representation of Malay and Indian youths in the “high difficulty” class suggests underlying structural or cultural factors that may exacerbate vulnerability. These factors include lower chronic disease and cancer screening rates (26), higher obesity and smoking prevalence (27, 28) and culturally influenced health behaviours among Malay and Indian communities, which have been identified in prior research as contributing to their greater vulnerability and poorer health outcomes in Singapore (29–31).

Recent projections from the Future Elderly Model (32) also indicate a rising prevalence of chronic conditions, comorbidities, obesity, and disabilities, with notable ethnic differences. Malay and Indian older adults are projected to face disproportionately higher rates of diabetes and hypertension by 2050, alongside a significant increase in comorbidities and disability prevalence compared to Chinese participants. These projections highlight the urgent need for early and targeted interventions to address long-term ethnic disparities in health outcomes even in the younger population (32).

Our finding that lower education attainment is associated with higher disability is consistent with Subramaniam et al. (33), who reported a higher likelihood of disability among Singaporean adults with lower education compared to those with a degree and higher education. Although we lack of data on chronic conditions and disability among youths in Singapore, our findings align with prior research on health disparities in adult and older adult populations (8, 16), confirming that ethnicity, education, and income are strong correlates of disability profiles. Lower educational attainment and lower household income also strongly predicted membership in higher difficulty classes, reflecting socioeconomic gradients in health and functioning. These patterns point to the intersection of disability with broader inequities in education and economic participation, which may perpetuate disadvantage across the life course if left unaddressed.

We also found that those belonging to the “high difficulty” class were more likely to have moderate and severe/very severe depression, as well as moderate and severe/very severe anxiety. These findings align with evidence that disability and mental health difficulties often co-occur in bidirectional and mutually reinforcing ways. Previous studies have consistently observed a significant association between depression and anxiety with disability (13, 20, 21, 31). Hendriks et al. (20) reported that all types of anxiety disorders such as generalized anxiety disorder, social anxiety disorder, panic disorder with agoraphobia were significantly associated with higher disability. Similarly, Park et al. (21) demonstrated a strong association between the DASS depression score and increased disability scores in the WHODAS physical, psychological, and environment domains among young people with ASD. It is also important to note that evidence supports a reverse causality, i.e., mental health conditions can also act as significant risk factors for disability. Multinational studies have demonstrated that depression and anxiety disorders predict future increases in disability and functional impairment (34–36). These findings underscore the importance of early mental health intervention as a strategy for preventing disability progression in youth.

We also found that individuals in the ‘high difficulty’ and ‘moderate social and functional difficulty’ classes have strong associations with various healthcare utilization with higher odds of hospital admission, A&E visits, contact with the polyclinic doctor and restructured hospital doctor in the past 3 months. We observed that the proportion of hospitalizations among youth in high difficulty class (5.4%) (**Supplementary Table 3**) was slightly higher as compared to the proportion of hospitalization among adult (2.3%), and older adult (4.7%) population in Singapore (37, 38). This may reflect higher treatment needs among this group or increased help seeking among youths who are less likely to stigmatise mental health conditions (39). However, despite increased contact with the healthcare system, it is unclear whether these youths are receiving timely and appropriate care for their functional and psychological needs. The findings highlight opportunities for early detection and intervention in primary care and emergency settings, where screening for both disability and psychological distress could be integrated into routine practice.

The present study has some limitations that must be considered while interpreting our results. First, data on disability, psychological distress, and healthcare utilization were based on self-report and the information may be influenced by recall and memory biases. Second, the cross-sectional design precludes causal inferences. Hence, caution must be exercised when interpreting our findings considering these limitations. Notwithstanding these limitations, the present study has notable strengths including its large, population-based sample and the usefulness of a person-centred analytical technique. Our study findings address important gaps in the literature, particularly by identifying distinct disability profiles among youths in multiethnic Asian populations.

## Conclusion

Disability among Singaporean youth is heterogeneous, comprising distinct profiles with different sociodemographic correlates and health outcomes. The strong connection between specific disability profiles, particularly those with high difficulties in all domains and moderate social and functional difficulty and both, psychological distress and use of direct medical care highlights a critical area for public health intervention. School- and community-based programmes may serve as critical platforms for early identification, stigma reduction, and provision of supports. At a systems level, the findings reinforce the importance of strengthening cross-sectoral strategies under Singapore’s Mental Health and Wellbeing Strategy, ensuring that services are inclusive of youths with both psychological and functional impairments.

## Data Availability

The raw data supporting the conclusions of this article will be made available by the authors, without undue reservation.
