# Supplementary material for: Profiles of disability among youths in Singapore and their link to psychological distress and health care utilization
Source: Front Psychiatry. 2026 Jun 1;17:1821187. doi: 10.3389/fpsyt.2026.1821187 (PMC13265559; doi:10.3389/fpsyt.2026.1821187)
Supplement: Supplementary file 1 [file SupplementaryFile1.docx]

Supplementary Tables

Table 1 Model comparisons of Latent Class Analysis

| Model | Log-likelihood | Parameter | AIC | BIC | Adj. BIC | Entropy | VLMR test | Lo-Mendell  Rubin  adjusted LR  test | Class proportion | | | | | |
| --- | --- | --- | --- | --- | --- | --- | --- | --- | --- | --- | --- | --- | --- | --- |
| LCA |  |  |  |  |  |  |  |  | 1 | 2 | 3 | 4 | 5 | 6 |
| Class 2 | - 8638.74 | 25 | 17327.47 | 17474.05 | 17394.62 | 0.956 | <0.001 | <0.001 | 0.16 | 0.84 |  |  |  |  |
| Class 3 | -8003.70 | 38 | 16083.39 | 16306.19 | 16185.46 | 0.893 | <0.001 | <0.001 | 0.72 | 0.08 | 0.21 |  |  |  |
| Class 4 | -7829.54 | 51 | 15761.09 | 16060.11 | 15898.07 | 0.899 | <0.001 | <0.001 | 0.14 | 0.07 | 0.08 | 0.72 |  |  |
| Class 5 | -7714.26 | 64 | 15556.53 | 15931.78 | 15728.43 | 0.893 | 0.6556 | 0.6578 | 0.06 | 0.04 | 0.06 | 0.72 | 0.12 |  |
| Class 6 | -7619.60 | 77 | 15393.20 | 15844.67 | 15600.02 | 0.900 | 0.6076 | 0.6093 | 0.72 | 0.06 | 0.02 | 0.04 | 0.05 | 0.12 |

Table 2 Item probabilities for the four-class model

|  | Class 1 –  High Difficulty  (n=244, 7.8%) | Class 2 –  Moderate Social and Functional Difficulty  (n=401, 13.8%) | Class 3 –  High Physical and Cognitive Difficulty  (n = 174, 6.7%) | Class 4 –  No/Low Difficulty (n=1781, 71.7%) |
| --- | --- | --- | --- | --- |
| Q1. Standing for long periods | 0.729 | 0.198 | 0.704 | 0.014 |
| Q2. Taking care of household responsibilities | 0.771 | 0.193 | 0.929 | 0.026 |
| Q3. Learning a new task | 0.795 | 0.188 | 0.720 | 0.015 |
| Q4. Joining in community services | 0.751 | 0.336 | 0.322 | 0.010 |
| Q5. Being emotionally affected by health problems | 0.769 | 0.398 | 0.403 | 0.027 |
| Q6 Concentrating for 10 minutes | 0.953 | 0.362 | 0.119 | 0.028 |
| Q7. Walking long distances | 0.912 | 0.194 | 0.080 | 0.007 |
| Q8. Washing whole body | 0.763 | 0.045 | 0.000 | 0.000 |
| Q9. Getting dressed | 0.789 | 0.036 | 0.008 | 0.000 |
| Q10. Dealing with unknown people | 0.928 | 0.493 | 0.045 | 0.034 |
| Q11. Maintaining a friendship | 0.893 | 0.394 | 0.041 | 0.011 |
| Q12. Day to day work | 0.897 | 0.447 | 0.157 | 0.028 |

Table 3 Proportion of healthcare utilization across class membership

|  | Hospitalization | A&E | Polyclinic doctor | Restructured hospital doctor | Private doctor |
| --- | --- | --- | --- | --- | --- |
|  | % (95% CI) | % (95% CI) | % (95% CI) | % (95% CI) | % (95% CI) |
| Disability subtypes |  |  |  |  |  |
| Class 1 – High Difficulty | 5.39 (2.9,9.83) | 8.49 (5.37,13.17) | 41.64 (34.74,48.89) | 19.93 (14.58,26.62) | 21.26 (15.8,27.99) |
| Class 2 – Moderate Functional  Difficulty | 3.68 (2.08,6.44) | 9.44 (6.8,12.97) | 39.56 (34.24,45.14) | 20.37 (16.32,25.12) | 28.03 (23.15,33.5) |
| Class 3 – High  Physical and Cognitive Difficulty | 1.38 (0.27,6.7) | 5.28 (2.47,10.92) | 30.77 (23.65,38.94) | 14.11 (9.22,20.99) | 32.74 (25.08,41.45) |
| Class 4 – No/Low Difficulty | 1.49 (0.96,2.3) | 3.33 (2.57,4.32) | 28.67 (26.38,31.07) | 12.01 (10.42,13.8) | 26.83 (24.5,29.3) |
